# Local Anæsthesia of the Larynx

**Published:** 1881-05

**Authors:** 


### LOCAL ANÆSTHESIA OF THE LARYNX.

To avoid the disagreeable effects of local anæsthetics hitherto employed in the larynx, Rossbach attempted to secure anæsthesia of the throat and larynx by the internal administration of large doses of bromide of potassium, and with right good results. But the loss of energy and the helplessness of the patient thus produced essentially interfere with the success of operative procedures. The author then endeavored to induce anæsthesia by interruption of the nerve force in sensitive nerves of the larynx. The trunk of the sensitive branch of the superior laryngeal nerve, at the spot where it penetrates the thyro-hyoid membrane below the button-shaped end of the great cornu of the hyoid bone, lies so superficially that it may be here completely anæsthetised by hypodermic injections of morphia 0.005 on each side. Still simpler and equally effective is the application of cold at these places. The cold is best brought to bear upon the spots by means of Richardson's atomisers which are so altered by Rossbach that the stream plays through two fine tubes directly upon the nerve trunks. Complete anæsthesia occurs after one or two minutes application of the atomizer.—*Wiener Med. Presse,* 1880, No. 40.—J. T. W.

